# Timing of Early Salvage Therapy for Patients With Biochemical Relapse of Prostate Carcinoma

**DOI:** 10.3389/or.2023.10676

**Published:** 2023-09-13

**Authors:** Soňa Argalácsová, Michal Vočka, Otakar Čapoun, Lukáš Lambert

**Affiliations:** ^1^ Department of Oncology, First Faculty of Medicine, Charles University and General University Hospital in Prague, Prague, Czechia; ^2^ Department of Urology, First Faculty of Medicine, Charles University and General University Hospital in Prague, Prague, Czechia; ^3^ Department of Radiology, First Faculty of Medicine, Charles University and General University Hospital in Prague, Prague, Czechia

**Keywords:** biochemical relapse, prostate cancer, high risk, salvage radiotherapy, timing

## Abstract

Between 25% and 33% of patients after radical prostatectomy experience a relapse of the disease. The risk of relapse increases in patients with risk factors up to 50%–80%. For a long time, adjuvant radiotherapy has been considered the standard of care. Four large prospective trials, that compared adjuvant and salvage radiotherapy in patients with biochemical relapse, showed the superiority of the adjuvant approach in biochemical and local relapse-free survival, but no consistent benefit in long-term endpoints (i.e., metastasis-free survival, overall survival, or carcinoma-specific survival) at the expense of increased urinary and bowel toxicity. Three large international studies comparing adjuvant and salvage radiotherapy paved the way toward early salvage radiotherapy. However, the optimal threshold of the PSA level (range of 0.2–0.5 ng/mL) for initiating early salvage radiotherapy remains unresolved and still poses a challenge in everyday clinical practice when balancing the need for early radiotherapy and the associated toxicity. Imprecise stratification of biochemical relaps patients according to the risk of clinical relapse drives efforts to find additional molecular biomarkers that would improve the timing of the salvage therapy.

## Introduction

Because of the large heterogeneity of prostate carcinoma (PCa) some patients may have an indolent disease without any progress in the patients’ lifetime while in others, the disease could have a very aggressive and lethal course [1]. Active surveillance is an optional approach for patients with very low- or low-risk PCa. This approach aims to distinguish patients with a latent, slow-progressing disease who would not benefit from any active therapy, from those with progressive disease, in whom early active treatment would prolong their survival. For localized PCa, therapeutic options include radical prostatectomy (RP) or definitive radiotherapy (RT). Both of them have comparable outcome but differ mainly in acute and late toxicity [2]. In 25%–33% patients after RP, relapse occurs later in the course of the disease [3, 4]. The risk of relapse after RP increases up to 50%–80% in patients with positive surgical margin (PSM), pT3a-extraprostatic extension (EPE), pT3b-seminal vesicle invasion (SVI), pathologically proven pelvic nodal involvement, and Gleason score (GS) 8–10/International Society of Urological Pathology (ISUP) Group 4–5 [5, 6].

## Risk Status After Radical Prostatectomy

### Adjuvant Radiotherapy as a Standard of Care

For a long time, adjuvant radiotherapy (ART) with or without androgen deprivation therapy (ADT) has been the standard of care for patients with the above-mentioned risk factors. The effects of ART have been studied in four major prospective trials (EORTC 22911, SWOG 8794, ARO96-02, FP-FINROG-0301), several smaller studies, and their meta-analyses [5, 7–10]. The benefit of ART has been consistently demonstrated only for biochemical relapse-free survival (bRFS) (hazard ratio - HR 0.47, *p* < 0.001) and locoregional relapse-free survival (lrRFS) (HR 0.54, *p* < 0.001). The studies and their meta-analysis also demonstrated higher overall toxicity (between 11% and 18% of patients) and severe genitourinary (GU) and gastrointestinal (GI) toxicity of ≥ Grade 3 (G3) by 1%–17% in the ART group compared to control arm [7]. The interpretation of the major four trials and their meta-analysis is complicated by inconsistent inclusion criteria, especially where staging and the requirement of a prostate-specific antigen (PSA) decline to zero after RP are concerned. The requirement of a zero PSA value after RP was implemented only in the ARO 96-02 study; the other three studies included 30%–70% of patients with PSA persistence [5, 7–10]. Besides, older techniques (2D or 3D RT) with nowadays insufficient doses (60 Gy) complicate the overall assessment of the results. Although the toxicity of multimodal therapy is significantly higher, neither individual studies examining the benefit of ART nor their subsequent meta-analysis have demonstrated a clear benefit of this therapy regarding overall survival (OS) and cancer-specific survival (CSS). The risk of overtreatment with ART is estimated by some authors to be as much as 35%–60% [7]. The results of another meta-analysis by Tao et al., which evaluated 15 smaller retrospective studies with a cumulative total of 5.586 patients, demonstrated a statistically significant benefit of ART versus salvage radiation therapy (SRT) in both 5 and 10 years bRFS and 5 years OS [4]. Based on these studies and their meta-analyses, the attitude towards ART and SRT remains inconsistent both among and within professional societies. While the European Society of Clinical Oncology (ESMO) no longer recommends ART as standard therapy, the recommendations of the European Urological Association (EAU) and the American Society of Clinical Oncology (ASCO), American Society for Radiation Oncology (ASTRO), and American Urological Association (AUA) remain more restrained in their recommendations and advocate considering ART after RP to patients with risk factors [2, 6, 11].

### Paradigm Shift From ART to SRT

Both ART and SRT aim to eradicate the microscopic disease that may lead to future macroscopic relapse. SRT, unlike ART, is indicated if PSA exceeds 0.2 ng/mL from postoperatively undetectable values (PSA-recurrence or biochemical relapse [BCR]; occurs in approx. 27% of patients after RP) or if PSA levels persist at ≥ 0.1 ng/mL for 4–8 weeks after RP (PSA-persistence, in 5%–20% of patients) [5, 6, 12]. The need for adequate assessment of the role of ART has been recently fulfilled with published results of head-to-head studies, comparing the effect of ART to that of SRT (RADICALS-RT, TROG 08.03/ANZUP RAVES, GETUG-AFU 17) and their meta-analysis (ARTISTIC) [3, 13–15]. The RADICALS–RT trial, conducted at several European, UK, and Canadian centers, randomized a total of 1,396 patients with risk factors for recurrence in a 1:1 ratio to groups with immediate ART after RP and a group referred for SRT when PSA rose again above 0.4 ng/mL. With a median follow-up of 4.9 years, the OS and metastasis-free survival (MFS) data have not been sufficiently mature at the time of publication. While this study reported a significant reduction in GU and GI toxicity due to SRT and improved patient-reported Quality of Life (QoL) in the SRT arm, it failed to show any difference in the 5 years bRFS (85% for ART vs. 88% for SRT, *p* = 0.56) [13]. The Australian TROG 08.03/ANZUP RAVES trial, which randomized 333 patients, demonstrated a 5 years bRFS survival in 86% of patients in the ART arm and 87% in the SRT arm, respectively, thus demonstrating non-inferiority of SRT (HR 1.12, p_*non-inferiority*_ = 0.15), along with a significant absolute reduction in GU toxicity ≥ Grade 2 (G2) by 16% [3]. The third study was the French GETUG-AFU trial 17, which randomized 424 patients to ART or SRT in conjunction with a 6 months ADT with triptorelin. This study found no significant difference in the 5 years event-free survival (EFS) (92% in the ART arm and 90% in the SRT respectively, HR 0.81, *p* = 0.42), again with the significant benefit of lower acute and late toxicity in the SRT arm (by approx. 20% absolute) [14]. The ARTISTIC meta-analysis confirmed no benefit in EFS (HR 0.95, *p* = 0.7) for ART, thus shifting the therapeutic paradigm towards early SRT [15]. Table 1 summarizes the characteristics and results of the main phase 3 studies with 1:1 randomization to ART and WW (EORTC 22911, SWOG 8794, ARO96-02, FP-FINROG-0301) and studies with randomization into ART and SRT arm (RADICALS-RT, TROG 08.03/ANZUP RAVES, GETUG-AFU 17).

**TABLE 1:** Characteristics of the principal prospective studies focusing on ART versus SRT.

	EORTC 22911 [9]	SWOG 8794 [8]	ARO 96-02 [5]	FP-FINROG-0301 [10]	RADICALS-RT [13]	TROG 08.03/ANZUP RAVES [3]	GETUG-AFU 17 [14]
Design	ART within 16W WW	ART WW	ART WW SRT for PSA persistence	ART within 12 W WW	ART within 26 W SRT within 2 M from BCR	ART within 6 M SRT 4 M from BCR non-inferiority	ART within 3–6 M SRT at BCR ADT for 6 M for all patients
Nr. pts.	1,005	425	307	250	1,396	333	424
mFU (years)	10.6	12.6	9.3	9.0	4.9	6.1	6.2
Main Inclusion criteria	≤75 Y old PS 0–1 WHO pT2-3N0M0 and ≥1 RF:ECE—77%SVI—25.5%.PSM—62.6%	PS 0–2 ≥1 RF: pT3a/b– 67%SVI—10% both– 22%p/cN0 M0 PLDN allowedno signs of incontinece	<76 Y old PS 0–1 WHO pN0 M0ECE—45%SVI—17% pT3c/pT4—36%PSM—68%**-** non-detectable PSA after RP	pT2N0M0 and PSM pT3a N0M0no SVI (pT3b)c/pN0M0	Postoperative PSA ≤0.2 ng/mL and ≥1 RF: pT3-4–76%PSM—63%GS ≥ 7–93% iPSA 10 ng/mL	PS 0–1 PSA ≤0.2 ng/mL and ≥1 RF:ECESVI—20%PSM—67%GS ≥ 7–97%	PS 0–1 pT3–pT4a ECE—77% SVI—21% GS ≥ 7–90% pNx (no PLND)/pN0(PLND)PSM
Endpoints	1^o^ bRFS	1^o^ MFS	1^o^ bRFS	1^o^ bRFS	1^o^ CSS	1^o^ bPFS	1^o^ EFS
	2^o^ LRFS, MFS, OS, CSS, toxicity, QoL	2^o^ bRFS, OS, QoL	2^o^ MFS, OS, toxicity, QoL	2^o^ OS, CSS, toxicity	2^o^ FFDM, OS, bPFS, toxicity, QoL	2^o^ bRFS, OS, QoL	2^o^ MFS, OS, toxicity, QoL
RT	60 Gy/30 fr. 2D-RT	60–64 Gy/30–32 fr., 2D-RT	60Gy/30 fr. 3D-RT	66.6 Gy/37 fr., 3D-RT	66Gy/33 fr. or 52.5Gy/20 fr.	64 Gy/32 fr. RT-PLND and ADT not allowed	66Gy/33fr.
					RT-PLND allowed (7% ART, 3% SRT)		RT-PLND allowed
					ADT allowed: (24% ART, 27% SRT)		ADT (triptorelin 6M) allowed (18% ART and 24% SRT)
PSA after RP > 0.2 ng/mL	29.9%	33%	**0%** in the ART	ART: 70%; WW: 65%	NA	NA	NA
	Pre-RT PSA:						
	≤0.2: 70.3% ART						
	68.6% WW						
	>0.2: 28.7% ART						
	31.2% WW						
Definition of BCR (ng/mL)	>0.2	>0.4	>0.05	>0.4	>0.4 after RP or >2.0 ng/mL anytime	>0.4	>0.4 ng/mL within 6 M after RT or >1.0 ng/mL anytime
Rate of SRT in WW	41%	37%	NA for ART	86%	NA	NA	NA
Median PSA before SRT	1.7 ng/mL in WW-arm	0.75–1.0 ng/mL in WW-arm	NA for ART	0.7 ng/mL in WW-arm	0.2 ng/mL (0.1–0.3)	>0.2 ng/mL - 50%	>0.24 ng/mL - 54%
Mean time to SRT	2.9 y	33% immediately	NA for ART		32% 5y after RP	33% immediately	23 M (4–100)
		87% within 6 M			33% 8 y after RP	87% within 6 M	
		37% LR					
Results:							
bRFS	10 y ART 60.6% vs. SRT 41.1%	mFU ART 60.7% vs. SRT 47.4%	5 y ART 77% vs. SRT 54%	10 y ART 82% vs. SRT 61%	5 y ART 85% vs.	5 y FFBP ART 86% vs. SRT 87% (HR 1.12, *p* = 0.15)	5 y EFS: ART 92% vs. SRT 90% (HR 0.81, *p* = 0.42)
			10 y ART 56% vs. SRT 35%		SRT 88% (*p* = 0.56)	8 y FFBP ART 80% vs. SRT 77%	
OS	10 y ART 76.9% vs. SRT 80.7% (*p* = 0.3407)	mOS: ART 15.2 y vs. SRT 13.3 y (HR_ART_ = 0.72, *p* = 0.023)	5.5 y: ART 96.6% vs. SRT 95%	10 y ART 92% vs. SRT 87% (*p* = 0.4)	9 y ART NA vs. SRT 96%	5 y ART 99% vs. SRT 98%8 y ART 92% vs. SRT 97%	5 y ART 96% vs. SRT 99% (HR 1.60, *p* = 0.25)
		mFU ART 74% vs. SRT 66%	9 y: ART 86.5% vs. SRT 85.5%				
CSS	10 y	NA	NA	10 y	NA	NA	ART 99% vs.
	ART 96.1% vs. SRT 94.6%			ART 99% vs. SRT 99%			SRT 99%
MFS	10 y ART 89.9% vs. SRT 89%	mFUART 57% vs. SRT 46% (HR_ART_ = 0.71, *p* = 0.016)	5.5 y ART 98% vs. SRT 96.9%9 y ART: 84.3% vs. SRT 85.1%	10 y ART 98% vs. SRT 96% (*p* = 0.4)	9 y FFDM ART NA vs. SRT 9%	5 y FFLDP ART 96% vs. SRT 96% 8 y FFLDP ART 93% vs. SRT 91%	NA
Toxicity:							
Acute toxicity	GU ≥ G2: ART 21.3% vs. SRT 13.5%GI ≥ G2: ART 11% vs. SRT 4%	NA	GU ≥ G2: ART 2% vs. SRT 0%GI ≥ G2: ART 1.2% vs. SRT 0%	NA	GU < 2 years: Any G: ART 54% vs. SRT < 26% ≥ G3: ART 12% vs. SRT <7%	GU ≥ G2: ART 70% vs. SRT 54%GI ≥ G2: ART 14% vs. SRT 10%	GU ≥ G2: ART 17% vs. SRT 4% ≥ G3: ART 3% vs. SRT 2%
					GI ≥ G2: ART 2.5% vs. SRT 1.9%		GI < 2 years: Any G: ART 62% vs. SRT 25% ≥ G3: ART 2% vs. SRT <2%
Late toxicity	Any: ART 70.8% vs. SRT 59.7%≥G3: ART 5.3% vs. SRT 2.5%	Any: ART 23.8% vs. SRT 11.9%	Any: ART 21.9% vs. SRT 3.7%≥G3: ART 1% vs. SRT 0%	Any: ART 56% vs. SRT 40%	GU > 2 years: Any GU: ART 40% vs. SRT 19% ≥ G3: ART 9% vs. SRT <5%	NA	GU: Any: ART 73% vs. SRT 29% ≥ G2: ART 27% vs. SRT 7% ≥ G3: ART 6% vs. SRT 1%
					GI > 2 years: ART 33% vs. SRT 15% ≥ G3:ART 3% vs. SRT < 2%		GI: Any: ART 44% vs. SRT 20% ≥ G2: ART 8% vs. SRT 5% ≥G3: ART 4% vs. SRT <1%
Erectile dysfunction	NA	NA	NA	NA	NA	≥G2:	Any:
						ART 98% vs.	ART 36% vs.
						SRT 96%	SRT 13%
							≥G2:
							ART 28% vs.
							SRT 8%
							≥G3:
							ART 4% vs.
							SRT 1%

mFU, medical follow-up; Nr. pts., Number of patients; M, month; W, weeks; y, years; RP, radical prostatectomy; ART, adjuvant radiotherapy; WW, watch and wait/observation; SRT, salvage radiotherapy; BCR, biochemical relapse; RF, risk factors for recurrence; PS, performance status; ECE, extracapsullary extension; SVI, seminal vesicle infiltration; PSM, positive surgical margins; GS, Gleason score; PLND, pelvic lymph node dissection; PSA, prostate specific antigen; iPSA, initial PSA; ADT, androgen deprivation therapy; RT, radiotherapy; fr., fractions; bRFS, biochemical relapse-free survival; PFS, progression-free survival; LRFS, local relapse-free survival; MFS, metastasis-free survival; OS, overall survival; CSS, carcinoma-specific survival; EFS, event-free survival; QoL, quality of life; FFDM, Freedom from distant metastases; FFBP, freedom from biochemical progression; FFLDP, freedom from local and distant progression; LR, local relapse; GU, genitourinary toxicity; GI, gastrointestinal toxicity; NA, not applicable.

The bold value means no patient in the ART arm.

### Addition of ADT to SRT

The basis for combining SRT with ADT was laid by two main studies. The RTOG 9601 trial that compared survival in men with PSA persistence or recurrence (PSA 0.2–0.4 ng/mL) after RP and with high-risk factors for relapse, in whom SRT (64.8 Gy) was indicated with or without a 2 years ADT with bicalutamide, 150 mg/day. At a 13 years follow-up, the group with ADT addition demonstrated a statistically significant improvement in 12 years OS (76.3% vs. 71.3%, HR 0.77, *p* = 0.04) and an 8.5% reduction in 12 years mortality due to prostate cancer (14.5% vs. 23.0%, *p* = 0.005), with no other statistically significant increase in toxicity except for gynecomastia (67.9% vs. 10.9%, *p* < 0.001). A subgroup analyses revealed that the greatest benefit from the addition of ADT was observed in patients with pre-SRT PSA levels >0.7 ng/mL, GS 8–10, and PSM [16]. The French GETUG-AFU 16 trial compared the effect of the addition of short-term ADT with gosereline for 6 months to the SRT therapy (66 Gy) in 743 patients with high-risk factors for relapse and pre-SRT PSA of 0.2–2 ng/mL after RP. In that study, no statistically significant benefit for overall survival was demonstrated, nevertheless, a significant reduction in 5 years bPFS (80% vs. 62%, HR 0.5, *p* < 0.0001) as well as a reduction in MFS (HR 0.73, *p* = 0.034) was demonstrated in the ADT group with no significant increase in late toxicity. A 112 months follow-up demonstrated that the addition of short-term ADT leads to persistent reduction of biochemical progression 10 years bPFS (64% vs. 49%, HR 0.54, *p* < 0.0001) compared with SRT alone [17, 18]. Based on a retrospective analysis of 1,125 patients and the main factors of clinical recurrence (≥pT3b, GS ≥ 8 and pre-SRT PSA > 5 ng/mL), Fossati et al. recommended the administration of short-term ADT (for one risk factor, RF) or long-term ADT (for two or more RFs) [6, 19]. The results from a study by Dess et al. suggested that pre-SRT PSA level > 0.6 ng/mL should be a prognostic biomarker for OS-benefit of ADT administered with SRT [20]. Although the data available so far, do not prove the necessity of the addition of standard ADT to SRT, they suggest that it might be considered especially in patients with the abovementioned risk factors.

### Prognostic and Predictive Tools for SRT

In patients with PSA persistence, who have a worse prognosis compared to those with PSA recurrence, biochemical progression occurs in 50%–75%, but only about one-third of them develop distant metastases within 3 years of RP and almost 40% of them remain free of distant metastases even after 7 years [10, 12, 21–24]. Due to the aforementioned risk of overtreatment of ART, it has been suggested that approximately one–third to one-half of patients with PSA recurrence may not need RT at all or at least not so early considering all the consequences [7]. These assumptions are supported by sub-analyses of the RADICALS-RT study, according to which only one-third of patients in the SRT arm were indicated for therapy within 8 years of RP [7, 13, 24]. Results of other studies imply that only 30% of BCR patients manifest clinically and only 16% of them die of PCa progression [25–27]. Based on a meta-analysis of 77 studies with nearly 45,000 patients, the following clinical risk factors were identified as the primary negative prognostic factors for long-term survival: short PSA doubling time (PSA-DT), high postoperative GS, and short interval to biochemical relapse (IBR) [25]. The European Urological Association (EUA) has defined two risk groups for recurrence after RP—namely, a low-risk group (PSA-doubling time [PSA-DT] > 1 year and pathological GS < 8 and IBR > 18 months) and a high-risk group (PSA-DT < 1 year, pathological GS 8–10 and IBR < 18 months). Thus, the combination of parameters such as PSA-DT >1 year, BCR >3 years, stage ≤ pT3a, and ISUP grade 2/3 may help indicate postponing SRT while maintaining regular follow-ups [6, 25, 27]. Thus, all predictive tools that we have today, are based only on histological and biochemical parameters.

Advances in tumor-agnostic approaches in other cancers (e.g., melanoma or lung cancer), as well as the imperfect risk stratification leads to efforts to find molecular predictors that could, in combination with clinical factors, better stratify BCR patients according to the risk of clinical relapse, and thus help optimize therapy. These efforts have led to the development of three commercial multigene assays (Oncotype DX^®^ Prostate Cancer Assay Decipher®Test, Prolaris Cell Cycle Progression Assay), which differ significantly from each other. They have not entered routine clinical practice due to lacking validation on large patient population [28].

A meta-analysis of ten trials showed a significant improvement in 5 years bRFS in the group of patients with pre-SRT PSA < 0.5 ng/mL, compared with patients with pre-SRT PSA > 0.5 ng/mL [29]. By the pre-SRT PSA level of 0.5 ng/mL, the chance of re-achieving non-detectable PSA levels after SRT is approximately 60%; a chance for a 5 years RFS is as much as 80% [29, 30]. Abugharib et al. studied 657 patients with SRT and proved a strong correlation of pre-SRT PSA levels with the effect of early SRT. In their study, the groups with pre-SRT PSA of 0.01–0.2, 0.2–0.5, and >0.5 ng/mL, respectively, showed gradually worse 10y-bRFS (62%, 44%, 27%, respectively), MFS (86%, 79%, 66% respectively), and CSS (93%,89%, 80% respectively) with the increasing pre-SRT levels [21]. According to the recommendations, the most appropriate pre-SRT PSA level for initiating SRT is up to 0.4–0.5 ng/mL, as patients with PSA > 0.5 ng/mL are at higher risk of distant dissemination [2, 6, 21]. Nevertheless, the questions of the appropriate timing of early SRT as well as the necessity of ADT and its duration remain largely unanswered.

## Discussion

The purpose of our overview was to summarize contemporary approaches to the patients with high risk (50%–80%) of recurrence after RP. Published studies suggest that one to two-thirds of patients will benefit from subsequent ART or SRT. Because of the large inter- and intra-tumoral heterogeneity of PCa, it has been suggested that at least one-third of these patients may be spared further multimodal therapy and the resulting consequences such as GU and GI toxicity.

A randomized phase III trial ESTABLISH (NCT05232578) that may clarify the need for early salvage radiotherapy in patients with BCR after RP with high-risk factors for relapse in the “gray zone” of PSA value 0.2–0.5 ng/ml has been initiated in the Czech Republic. Current prognostic and predictive factors based only on clinical parameters (stage, GS, PSA, PSA-DT, IBR) are insufficient for accurate stratification of patients to multimodal therapy. Clear predictive and prognostic molecular genetic tests facilitating this stratification and the choice and timing of the therapy for an individual patient have not yet been established in clinical practice. It seems, that the interplay of clinical and molecular prediction could be the right key to an accurate patient-oriented therapy. The suitability or even necessity of initiating early SRT and its balancing with associated toxicity in the PSA range 0.2–0.5 ng/mL remains an unresolved “grey zone” that poses a challenge in everyday clinical practice.
